# TLR9 in MAFLD and NASH: At the Intersection of Inflammation and Metabolism

**DOI:** 10.3389/fendo.2020.613639

**Published:** 2021-01-29

**Authors:** Christopher R. Shepard

**Affiliations:** Avogadro Development Corp, New York, NY, United States

**Keywords:** non-alcoholic fatty liver disease, fibrosis, CpG DNA, mitochondrial DNA, PPAR, AMPK, adiponectin, NF-kappa B

## Abstract

Toll-Like Receptor 9 (TLR9) is an ancient receptor integral to the primordial functions of inflammation and metabolism. TLR9 functions to regulate homeostasis in a healthy system under acute stress. The literature supports that overactivation of TLR9 under the chronic stress of obesity is a critical driver of the pathogenesis of NASH and NASH-associated fibrosis. Research has focused on the core contributions of the parenchymal and non-parenchymal cells in the liver, adipose, and gut compartments. TLR9 is activated by endogenous circulating mitochondrial DNA (mtDNA). Chronically elevated circulating levels of mtDNA, caused by the stress of overnutrition, are observed in obesity, metabolic dysfunction-associated fatty liver disease (MAFLD), and NASH. Clinical evidence is supportive of TLR9 overactivation as a driver of disease. The role of TLR9 in metabolism and energy regulation may have an underappreciated contribution in the pathogenesis of NASH. Antagonism of TLR9 in NASH and NASH-associated fibrosis could be an effective therapeutic strategy to target both the inflammatory and metabolic components of such a complex disease.

## Introduction

Non-alcoholic fatty liver disease (NAFLD) is the most common cause of liver disease worldwide (1). NAFLD is a progressive condition most closely associated with overnutrition. Obesity is an overwhelming condition for normal biological processes. The simplest distillation is that when lipid input exceeds the body’s ability for lipid disposal, a number of physiological stresses are put on the organs under metabolic overload: including the liver, the adipose compartment, and the gut. How the physiological stresses manifest disease is acknowledged as complex, and recently the term metabolic dysfunction-associated fatty liver disease (MAFLD) was recommended instead of NAFLD as a more appropriate overarching term for a disease that is a multifaceted confluence of metabolic and inflammatory processes (2, 3).

Non-alcoholic steatohepatitis (NASH) is the more progressive form of the disease that includes hepatocellular injury in addition to hepatic steatosis and mild inflammation. NASH may progress to hepatic fibrosis, which is, by far, the most critical determinant outcome for a course of disease at risk of progression to cirrhosis and hepatocellular cancer. NASH is an unmet medical need still lacking any approved therapeutics (4–6).

Toll-Like Receptor 9 (TLR9) is a pattern recognition receptor, so-called because this family of receptors is activated by recognizing common structures or motifs across biological molecules (7). There are at least ten TLRs present in mammals responsible for cell-extrinsic and -intrinsic activities (8, 9). Characterization of the common motifs and their physiological association have spawned several names and their associated acronyms, such as pathogen-associated molecular patterns (PAMPs), associated with microorganisms; damage-associated molecular patterns (DAMPs), associated with endogenous molecules released from damaged cells; and metabolism-associated molecular patters (MAMPS), associated with danger molecules resulting from metabolic overload (10, 11).

TLR9 detects the “CpG” motif, unmethylated cytosine-phosphate-guanine (CpG) dinucleotides. CpG is a common motif in bacterial and viral DNA, but uncommon in the vertebrate genome. However, mitochondria, the energy center of every vertebrate cell, is an organelle of bacterial origin and has its own mitochondrial DNA (mtDNA). Mitochondria are the source for endogenous molecules that activate TLR9 (12). Upon cell stress or damage, mtDNA is ejected from the mitochondria and into the cell’s surrounding environment to activate TLR9 as paracrine or endocrine signals for danger or stress.

## Discovery of TLR9 and the Trajectory of Subsequent Research

The breadcrumbs that led to the discovery of TLR function, and eventually TLR9, are relevant because they are important in the evaluation of TLR9 in NASH. O’Neill et al. have conducted comprehensive work in summarizing the research history of TLRs (13), of which a small slice will be focused on here to uncover the core biological functions of TLR9.

The discovery of the interleukin-1 (IL-1) family of molecules, called *necrosin* by Menkin in his original characterization in 1943 because of the observed inflammatory tissue injury (14), opened the door to the signaling of the innate immune system. By 1975, the connection between innate immunity and metabolism had been made with the molecular players still needing to be identified (15). Infection of rabbits with various bacterial pathogens caused marked changes in lipid levels (16), and carbohydrate-regulating hormones triggered by the host response were associated with biochemical and ultrastructural changes in the liver (17).

The soluble signaling molecules of the IL-1 family were characterized in seminal work at Harvard Medical School by Dayer, Krane and Robinson *et al*. and Mizel and Mergenhagen at the National Institutes of Health in the last years of the 1970s (18, 19). The receptor for those signaling molecules, IL-1R, was cloned in 1988 by Sims *et al*. while at Immunex, a private biotechnology company eventually acquired by Amgen. In that 1988 report, the authors write, “How the cytoplasmic domain functions in signal transduction is unknown. Computer searches of the 1987 edition of Genbank [and other databases] have no revealed significant similarity to any currently available sequences” (20). Research on the role of IL-1 signaling in inflammation continued concurrently with investigations into the effects on energy balance. Particularly prescient was a report by Kitade et al. in 1996 that IL-1b regulates metabolism in hepatocytes (21).

The discovery that launched the dissection of TLR signaling in mammals was catalyzed by a short communication in *Nature* in 1991 from the Department of Biochemistry at the University of Cambridge that the cytoplasmic domain of IL-1R was homologous to the cytoplasmic domain of the *D. melanogaster* Toll protein, which was only studied at the time in the development of dorsoventral polarity in fruit flies (22, 23). The shared domain came to be known as the Toll/interleukin-1 receptor (TIR) domain.

In 1996, when it was discovered that *Drosophila* does use Toll for immunity, a kind of parallax was created that moved Toll’s involvement in immunity to the forefront of research (24). Discovery of the human TLR homologs starting in 1997 occurred on this backdrop, and although no function was yet ascribed to the mammalian TLR, immunity seemed the most likely function (25). Recentered efforts by researchers on innate immunity minimized research efforts devoted to TLR’s potential involvement in energy balance. Relative to functioning in immune processes, TLR’s role as a mediator of metabolism became largely overlooked. TLR4 was identified as the signaling receptor for LPS in 1998 (26). TLR9, including its sub-family members TLR7 and TLR8, were cloned in 2000 with CpG-DNA identified as a ligand for TLR9 in the same year (27–30). The next decade of research swiftly confirmed the TLR family’s undoubted importance in immunity, inflammation, and involvement in autoimmune diseases.

It was not until 2009 that the connection was made in *Drosophila* that Toll pathway activation in the insect compartment analogous to the human liver leads to inhibition of insulin signaling that results in decreased triglyceride storage (31). In the human system, only a handful of publications in the intervening years have focused on the relationship between TLR9’s signaling, metabolism, and energy expenditure.

In the mid-2000s, IL-1b and TLR9 adaptor protein MyD88 were implicated in weight loss-associated activation of the innate immune receptors independent from the inflammatory cascade and associated inflammatory pathology, however the mechanism was unknown (32). A seminal paper on TLR9 signaling was published in 2010 that reported the bifurcation of TLR9’s signaling pathway into pro-inflammatory and non-inflammatory pathways (33). Three years later, TLR9 was directly implicated in modulating energy metabolism independent of its proinflammatory function (34). It was not until 2020 that TLR9 was found upstream of a master regulator of energy homeostasis, AMPK activation (35), a topic to which this review will later return. The observations of TLR9’s importance in regulating metabolism are consistent with the discoveries that the signature signaling domain of TLR9, TIR, is a primordial metabolic regulatory enzyme (36, 37).

**Figure 1:**
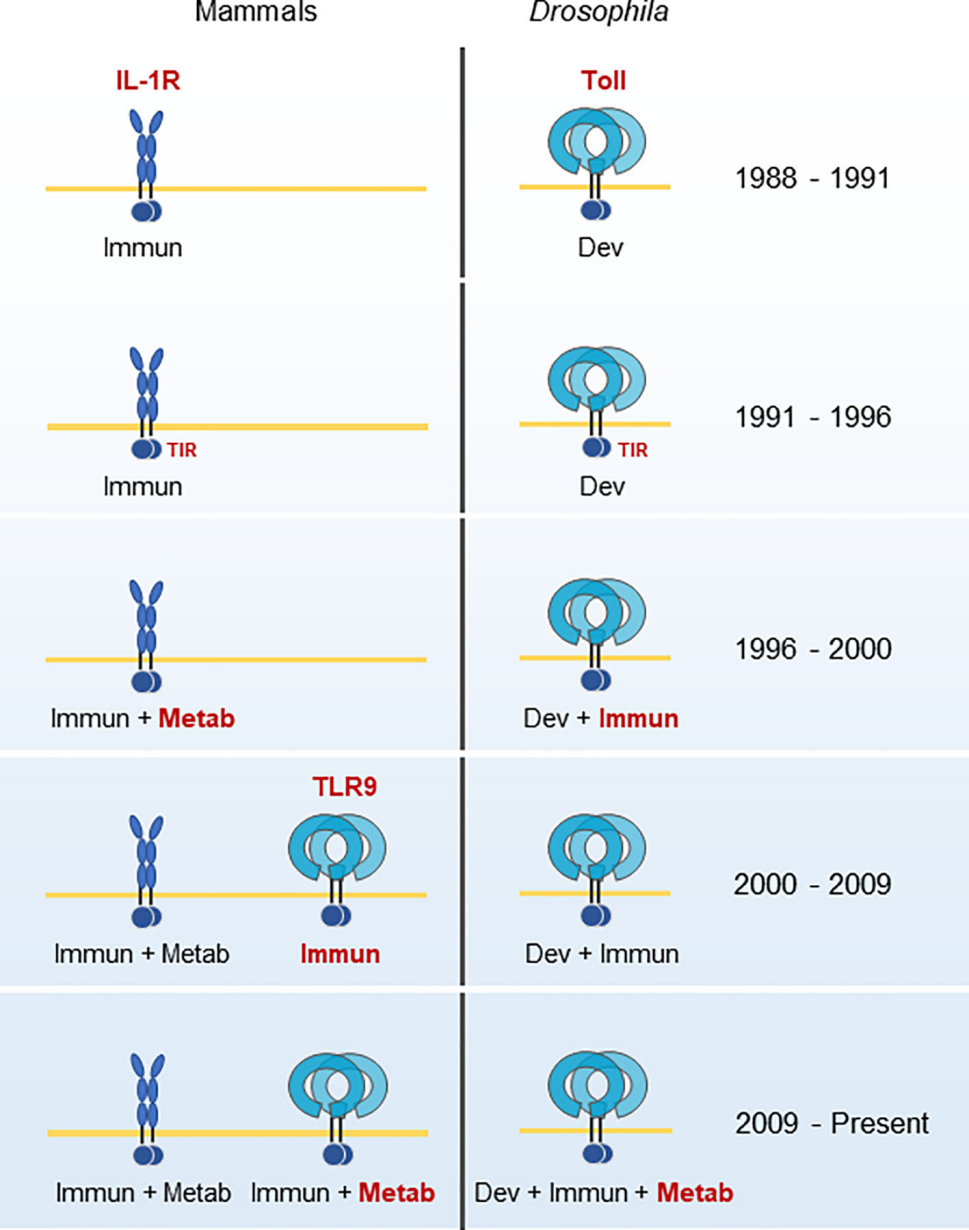
Historical developments in the TLR9 field. IL-1R’s role in immunity was known during the same period as Toll’s role in Drosophila development. The homology of their common signaling domain, TIR, was discovered in 1991. In 1996, a role for hepatocyte metabolic regulation for IL-1R and Toll’s role for immunity was discovered in *Drosophila*. TLR9 was cloned and identified as the immunological pattern recognition sensor for CpG oligonucleotides in 2000. It was not until 2009 that Toll and TLR9 were found to have direct roles in metabolism and energy homeostasis. Adapted from a figure by Beutler (38). Immun, Immunity; Dev, Development; Metab, Metabolism.

The role of TLRs as the gateway to the innate immune system is certain, and many articles summarize the history of this research in detail (13, 24, 38, 39). The slice of history presented here illustrates that TLR9’s role in metabolism is a relatively recent discovery, and perhaps underappreciated. The majority of research on TLR9 has investigated its function as an immune receptor (**Figure 1**). In the next section, we will find that TLR9 is an ancient protein. There is only one other primordial function as important as immunity, and that is metabolism.

## TLR9 Is an Ancient Receptor That Is Part of a System of Tiered DNA Sensing

TLR9 is one component of a tiered system of DNA pattern sensing by the cell (40). Emming and Schroder do an excellent job of offering a unifying hypothesis of the tiered structure that includes the endosomal TLR9, cytosolic or nuclear cGAS-STING, and cytosolic AIM2, all of which transduce inflammatory signals according to the level of threat. TLR9 functions as the canary—the leading signaling component for what is happening in the environment outside the cell. This system, or at least components of the system, appears throughout the tree of life (41). It is suspected that non-TLR9 elements evolved the ability to sense DNA relatively recently (42). The metabolic effects of DNA sensing are also a common feature even by extracellular sensing of DNA in protozoa and insects (43).

TLR9 has the lowest genetic drift of any of the components of the nucleic acid-sensing system and any TLR in mammals (41, 44–47). It is speculated that more intense selective pressures on other pattern recognition receptors may have left TLR9 intact (48).

All of this is to say that the extracellular sensing of DNA by TLR9 is a primordial program in response to danger. The group that initially characterized TLR9 as a sensor of bacterial DNA hypothesized that its specific action was to distinguish bacterial DNA from self-DNA (27). Polly Matzinger at the NIH urged the research community to change their point of view: she hypothesized that the primary driving force of the immune system is not to discriminate between self and non-self, but rather to protect and detect against danger (49). That TLR9 expression and upregulation are reported in nonimmune cells, including cardiomyocytes, neurons, hepatocytes, adipocytes, endothelial and epithelial, is suggestive of a critical role beyond immunity (50–55).

One of the foremost dangers of the 21^st^ century to population health is overnutrition that disturbs metabolic homeostasis. The clinical manifestation is the epidemic of obesity and the metabolic syndrome. On the physiological level, it is the insult of chronic positive energy balance that stresses those metabolic organs most involved in nutrient handling: the liver, adipose tissue, and gut. Connor et al. describe the blurred line in obesity between pathological and protective mechanisms in the cell (56). It has been known that diets enriched with lard prime the immune system (including TLRs) for response (57).

In TLR9’s role as a metabolic regulator, and more generally as a sensor of danger, surely overnutrition was not a selection pressure in its evolution in man. The remaining content of this article will review the investigation into TLR9 in MAFLD and NASH.

## TLR9 Expression

TLR9 is expressed in a broad range of immune cells with varying ability across tissues in the normal physiological state to respond to CpG nucleotides (58, 59). There is some evidence of differential expression of TLR9 isoforms across healthy immune-cell rich tissues and peripheral blood cell types with limited data that the isoforms may differ in cellular localization (60). The classical pro-inflammatory role of TLR9 is through the activation of NF-κB. Results from clinical survey studies and more focused basic research investigations suggest that TLR9 drives expansion of resident and patrolling monocytes and monocyte-derived dendritic cells in inflammatory diseases.

TLR9 expression in non-immune cell types is curious. Non-immune cells, such as hepatocytes and adipocytes, can produce inflammatory molecules, but not at similar levels to immune cells (61, 62). Similarly, the tissue cell-type-specific responses to TLR9 agonists is also conserved in non-immune cells. For example, in a rodent model of renal-ischemia reperfusion that produces mtDNA release from stressed cells, TLR9 on hepatocytes respond to the CpG mtDNA, while proximal tubular epithelial cells do not—despite both having increased TLR9 expression (52). These cell and tissue regulatory systems make sense, as it could result in a catastrophic inflammatory response if tubular epithelial cells responded to stress and danger signals the same as immune cells. TLR expression in non-immune cells could be serving another purpose—TLR activation in adipose tissue and liver in inflammatory states leads to the downregulation of metabolism-related genes (63).

TLR9 is normally expressed in organs of the alimentary tract, including the liver and intestines. Expression is higher in the small and large intestine than in the liver, a pattern that exists across normal human, conventional CD-1 mice, and C57BL/6 germ-free mice (64). There is a degree of tolerance in TLR9 signaling in the intestinal epithelium, which one would anticipate given the organ’s constant exposure to bacterial products. The tolerance is maintained through differential apical and basolateral signaling of TLR9 (65, 66). When intestinal epithelial cells are exposed to pathogenic DNA *in vitro*, TLR9 mRNA is upregulated and increases in surface localization (67). The resulting transient increase in TLR9 signaling until the danger has passed likely maintains cellular and physiological homeostasis. For example, that intestinal FXR is modulated by TLR9 should not be a surprise, but more studies are needed to resolve regulation in normal physiological conditions versus TLR9-mediated FXR downregulation in chronic inflammation (68, 69).

TLR9 is also expressed in two less obvious compartments important to the innate immune system: adipose tissue and the liver. Several observations were made in the early 2000s about the involvement of macrophages in adipose tissue [a good summary of the studies from this period is contained in the introduction in Cinti et al. (70)]. In lean populations of both rodents and humans, adipose tissue macrophages are M2-polarized (71, 72). It was a surprise in 2007 when TLR9 was also detected on cultured adipocytes (55), though at the time it was already known to be a functional immunological organ, secreting a variety of proinflammatory cytokines and modulating monocyte and macrophage function through adipokines (73).

The liver is a metabolic organ, and also the first line of defense against infection (74–76). Not only does one need to consider the functional cells of the liver, hepatocytes, but also the supporting architecture (sinusoidal endothelial cells) and the mix of resident immune cells (Kupffer, the resident macrophage of the liver; dendritic, and stellate cells). Hepatocytes have low TLR9 protein expression levels compared with the other resident immune cells of the liver (52). Functional TLR9 is undoubtedly expressed in the nonparenchymal cells of the liver, which comprise around 40% of the organ’s cells (77–80). TLR9 expression is similar in the livers of conventional (CD-1) mice and humans, with germ-free (C57BL/6 strain) mice having lower levels of expression (64). Interesting is the significantly greater expression of TLR7, a TLR9 family member that is both an RNA and CpG DNA pattern recognition sensor, in the mouse liver compared with humans. The minimal sequence requirement for activation of mouse-TLR9 and human-TLR9 is also slightly different, but the physiological consequence, if any, is unclear (81). Such species-specific differences are important in translating TLR-focused biology to humans.

TLR9 protein expression is dynamic. In resting cells, TLR9 is localized to the endoplasmic reticulum, and the shuttling to initiate signal transduction is complex (82). In cells stimulated with bacterial DNA, TLR9 mRNA expression increases and endosomal TLR9 protein is shuttled toward the surface (67). The upregulation in TLR9 gene expression also occurs with cell exposure to LPS (83).

TLR9 upregulation in organs with relatively low basal levels is also observed in systems stressed with chronic obesity. TLR9 is significantly increased in visceral compared to subcutaneous adipose tissue in non-diabetic obese patients (84). It should be noted that most of the expression comes from the stroma-vascular cell fractions of the compartment (e.g., adipose tissue macrophages), not the adipocytes. In liver, Geoffrey Farrell’s lab at the Australian National University found that TLR9 expression is significantly upregulated in patients with biopsy-verified NASH, but not in liver with bland steatosis (85). This result was similar to the upregulation of TLR9 in two other mouse models of NASH: atherogenic-diet fed and *foz/foz* mice. TLR9 expression was observed primarily in aggregates of inflammatory cells, but expression was also observed in binucleate cells that were most likely hepatocytes.

In the severely obese, the chaperone protein UNC93B, required for successful TLR9 trafficking, is also upregulated (86, 87). The upregulation of TLR9 and its chaperones in concert in obesity suggests an organized effort by the cell for increased TLR9 signaling.

## Signal 0 in NASH

Charles Janeway coined the term “Signal 0” in 1989 to describe non-antigen stimuli outside the sequence of steps (“Signals 1, 2, and 3”) that lead to adaptive immunity (88). Sterile inflammation, which occurs when DAMPs are released into the microenvironment, is a Signal 0 and prototypically underlies NASH.

Circulating mitochondrial DNA (mtDNA) is the principal suspect for sterile inflammation in NASH, allowing these danger signals to reach remote organs through the circulation. Various biological features contribute to normal basal fluctuations of circulating cell-free DNA in humans (89). While acute trauma, liver injury, or even strenuous exercise can significantly raise levels of circulating mtDNA temporarily (89–94), significant chronically elevated levels of circulating mtDNA exist in patients with obesity and T2DM (95, 96).

mtDNA copy numbers in subjects with metabolic dysfunction are also significantly increased in adipose tissue compared with patients with normal BMIs (97). Bariatric surgery significantly reduced elevated urinary mtDNA copy number in patients with obesity (96). The elevated levels of circulating mtDNA in patients with metabolic dysfunction is biologically meaningful. Elevated mtDNA levels were associated with IL-1b levels in patients with T2DM in two different studies (98, 99). Plasma concentration of cell-free DNA was significantly higher in patients with visceral adiposity and positively correlated with visceral fat area and insulin resistance (100).

Obese patients with liver dysfunction or patients with biopsy-confirmed NASH have elevated levels of circulating mtDNA. The observation of elevated circulating levels of mtDNA in obese patients who also had elevated ALT as a marker of chronic liver injury was first made by Wajahat Mehal’s group at Yale in 2016 (101) with preliminary data reported at the meeting of the American Association for the Study of Liver Diseases two years earlier (102). They reported that obese patients with ALT elevations had a greater percentage of mitochondria inside microparticles than lean subjects, and the fraction of microparticles containing mitochondria was also larger in obese subjects with ALT elevations. They determined that the microparticles were of hepatocyte origin.

Mehal’s group linked their observations with a previous finding that oxidized DNA increases the CpG motif-dependent response of TLR9 (103). They found a significantly larger fraction of oxidized DNA in the microparticles from plasma of obese subjects with ALT elevations. In circulating mitochondria not contained in microparticles, a fraction with an origin that could not be traced, they did not see similarly elevated levels of oxidized DNA. The group noted that the hepatocyte origin of the fraction of plasma microparticles containing mitochondria with increased oxidation is consistent with the observation of increased oxidation of hepatocyte DNA in NASH (104). Indeed, the microparticles could activate TLR9 in a reporter cell line.

In a different study, it was also demonstrated that *in vitro* treatment of hepatocytes with palmitic acid, to model lipotoxic overload, causes the release of mtDNA into the cytosol (105). Incubating the cultured hepatocytes with a superoxide scavenger prevented the release of mtDNA. Lipotoxic overload in hepatocytes is also known to impair autophagy, which may function in the progression from bland steatosis to NASH (106). shRNA-mediated knockdown of a master regulator of autophagy, BECN1, enhanced release of mtDNA into hepatocyte cytosol. Similarly, rapamycin, an inducer of autophagy, attenuated mtDNA release. Bafilomycin A1, an inhibitor of autophagy, enhanced mtDNA release. Therefore, mtDNA is intimately connected to hepatocyte fate.

The presence of elevated circulating mtDNA levels in NASH was validated in a later study by Yury Popov’s group at Harvard in 2020, who reported preliminary results at the meeting of the American Association for the Study of Liver Diseases three years earlier (107, 108). Rather than the relative fold-induction qPCR technique Mehal previously used to determine elevated levels of circulating mtDNA in cases of presumptive NASH, Popov determined absolute circulating mtDNA copy number in patients with biopsy-proven NASH and NASH-fibrosis. In separate pilot and validation cohorts, the group demonstrated that patients with biopsy-confirmed NASH had significantly elevated circulating mtDNA levels compared with healthy control patients. In the larger validation cohort of 114 samples, the group reported statistically significant elevations in mtDNA copy number in patients with NAS of 4 or greater compared with patients with NAS of less than 4 (p = 0.0334). With respect to fibrosis stage, patients with F2-4 had significantly elevated mtDNA compared with F0-1 patients (p = 0.0003). Based on the means and standard deviations from Popov’s source data, only about 20% of NASH patients (patients with NAS of 4 or higher) have levels of circulating mtDNA that are less than the 99^th^ percentile of circulating mtDNA in healthy patients.

Mehal and Popov’s results were further validated in a report from China, also in 2020 (105). This report is a valuable addition to the literature, as it has been shown that Chinese individuals have higher body fat percentages than Caucasians controls for any given BMI, with attendant increased visceral adiposity. Compared with other ethnic backgrounds, Chinese patients are affected by NAFLD at a lower BMI (109, 110). If chronic mtDNA elevation is a product of adipose tissue stress from overnutrition, one should see elevated circulating mtDNA levels in this population at lower BMIs. Indeed, Gao et al. observed elevated plasma levels of mtDNA in patients with BMIs in the lower 20s, consistent with healthcare professionals adopting a lower BMI threshold for clinical assessment and referral of NAFLD/NASH in these patients (111). In this relatively small report of 61 patients, divided into healthy control, NAFL, borderline-NASH (not defined in the paper, but generally defined as patients with an NAS of 3 (112)), and NASH, Chinese patients with borderline-NASH and NASH had significantly higher levels of mtDNA compared to healthy controls and non-NASH subjects.

Other diseases associated with elevated circulating mtDNA and NASH, independent from obesity, are intriguing. Sarcopenia, a muscle-wasting disorder associated with myocyte-specific mitochondrial dysfunction and circulating mtDNA, is associated with NAFLD independently of obesity and insulin resistance (113–116). The skeletal muscle compartment’s putative involvement in NASH disease deserves further exploration, as the inflammatory paracrine loop involving TLR9 in the sarcopenic muscle compartment may have analogs in other metabolic compartments (117–119). A similar pattern of NASH without metabolic risk factors exists in patients with HIV infection independent of combination antiretroviral therapy that causes liver damage (120, 121). Elevated adipose mtDNA levels are observed in HIV patients not on antiretroviral therapy, and elevated circulating mtDNA in HIV infected patients is a common observation (122, 123). One could hypothesize the presence of elevated circulating mtDNA levels links sarcopenia and HIV to attendant cases of NASH without the observation of obesity or metabolic dysfunction.

## TLR9 Drives NASH Pathogenesis Across Multiple Organ Systems

### Gut

The gut supplies the liver with the majority of its blood, which is likely a source for TLR9-activating molecules into the portal circulation. Richard Flavell’s laboratory tested the hypothesis that gut microbiota has a central role in the pathogenesis of NASH (124). They demonstrated that TLR9 is necessary for mice to be susceptible to NASH when they are co-housed with mice that harbor transmissible colitogenic gut microbiota. In contrast, TLR5, a receptor for bacterial flagellin, did not have an effect in mediating disease severity. In the same rodent model, they found that TLR9 agonist influx into the portal circulation increased the severity of NASH. Finally, they demonstrated that microbiota-dependent, subclinical inflammation of the colon caused by the induction and secretion of CCL5 by the colonic epithelium was significantly associated with the influx of TLR9 agonist into the portal circulation.

In contrast, there was no difference in the amount of TLR2 agonists, which encompass a wide range of microbial cell wall components from Gram-positive and Gram-negative bacteria, in the portal circulation of mice with colonic inflammation. That TLR9 agonists are elevated, but not TLR2 agonists, is consistent with the immune system killing and disintegrating microbes with the release of microbial nucleic acids (125–129). The data indicate that bacterial products derived from the intestine, most likely microbial cell-free DNA, enter the hepatic portal circulation and trigger TLR9 activation.

Translocation of bacterial contents from the gut may only be relevant in a subset of NASH patients. A meta-analysis of 128 NAFLD patients across five single-center studies employing the same intestinal permeability assay found that 39.1% of NAFLD patients had evidence of increased intestinal permeability compared with 6.8% of healthy controls (130). In the same meta-analysis, it was found that 49.2% of patients with NASH had increased intestinal permeability. The data in total suggest that alterations in intestinal permeability in at least a significant subset of NAFLD and NASH patients may contribute to liver injury *via* TLR9 activation.

### Liver

In a collaboration between Akita University and UCSD with Bernd Schnabl, Jerrold Olefsky, David Brenner, and Ekihiro Seki, Miura et al. first published on NASH in TLR9 knockouts (KOs) motivated by their interest in the TLR-MyD88 pathway in 2010 (131). They reported that TLR9^-/-^ mice showed less steatohepatitis and liver fibrosis than wild-type (WT) mice when both were fed a choline-deficient, L-amino acid-defined (CDAA) diet. Later studies revealed that mice on a CDAA diet display a significantly different metabolic profile from human NASH, and therefore this diet model is not suitable as the surrogate for human disease (132). Nonetheless, the severity of the inflammatory profile produced by this diet yields intriguing insights into the inflammation-related biology associated with NASH pathogenesis

The group demonstrated that Kupffer cells produced the inflammatory cytokine IL-1β in response to CpG oligonucleotides. IL-1β increased apoptosis and necrosis in lipid-accumulated hepatocytes isolated from mice fed the CDAA diet, while CpG-mediated TLR9 signaling had little effect on cell death in either normal or lipid-accumulated hepatocytes. They next examined IL-1β’s effect on the fibrogenic response of hepatic stellate cells (HSC). While numerical increases in the levels of molecular markers of fibrogenic activation when HSC were treated with CpG DNA were observed, IL-1β increased the same markers by a significantly larger fraction. Employing experiments with conditioned media from Kupffer cells, their conclusion was TLR9-mediated IL-1β release from Kupffer cells was essential for HSC activation. They also found that a knockout of MyD88, the adaptor molecule shared by TLR9 and the IL-1β receptor IL-1R, is crucial for signaling that promotes NASH and fibrosis. TLR9 knockout mice on the CDAA diet, unlike the IL-1R and My88 knockout mice on the same diet, had a significant reduction on HOMA-IR.

Therefore, Miura et al.’s model involves the TLR9-dependent activation of Kupffer cells through endogenous TLR9 ligands, which in turn stimulates IL-1β release that acts on both hepatocytes and stellate cells. The model is consistent with observation of increased IL-1β in patients with NASH and fibrosis (133, 134); increased number of IL-1β positive liver cells in mice fed a high fat and cholesterol diet (135); that the lack of IL-1β inhibits the transformation of steatosis to steatohepatitis (136); and that deficiency of hepatic- rather than bone marrow-derived IL-1β protected mice against development of steatohepatitis and liver fibrosis on an atherogenic diet (136). Also consistent with Miura et al.’s model is interventional data in humans with the IL-1β neutralizing antibody canakinumab in a population with high cardiovascular risk who were borderline obese. Canakinumab tempered metabolic-related inflammation, but it did not affect plasma lipoprotein levels or new-onset diabetes (137, 138). Therefore, while IL-1β therapy in NASH subjects has yet to be tested, the data suggests it may not be a node that significantly impacts both the inflammatory and dysfunctional metabolic components of the disease.

Wajahat Mehal’s group at Yale continued the investigation into the identities of the TLR9 ligands and NASH pathogenesis with a report in 2016 (101). Mehal’s group used a high-fat diet (HFD) model (45% fat), which results in a metabolic and histological profile similar to human NASH (139). Mehal’s work with a HFD was key to bridging previous work to produce results considered more translationally relevant in humans. The group’s experiments that led them to focus on hepatocyte mtDNA as the critical TLR9 ligand in this system was described in the Section titled *Signal 0 in NASH*. The determination of whether TLR9 is signaling to NF-κB or IRF-7 dependent type 1 IFN is key to determining TLR9’s role in the pathogenesis of NASH. While the paths are not mutually exclusive, the NF-κB pathway results in the production of proinflammatory cytokines, while the IRF-7 pathway can upregulate the anti-inflammatory IL-1 receptor antagonist (IL-1RA). mtDNA from hepatocytes fed a HFD, when cultured with primary murine macrophages, resulted in selective upregulation of pro-inflammatory cytokines, but not type 1 IFN. The group confirmed hepatic macrophage (Kupffer cell) NF-κB activation *in vivo* using an NF-κB reporter mouse. HFD for twelve weeks induced the upregulation of NF-κB on cells excised from liver tissue with macrophage markers.

The group generated mice in which TLR9 was selectively removed from lysozyme producing cells (Lysm-Cre Tlr9^fl/fl^), including neutrophils, monocytes, and tissue macrophages. WT, Lysm-Cre Tlr9^fl/fl^ mice, and total TLR9 knockout mice were placed on a HFD. The three groups had no difference in food intake as determined by paired-feeding experiment. The wild type mice on a 12-week HFD developed hepatosteatosis, balloon cells, and inflammation with elevated ALT, while both the Lysm-Cre Tlr9^fl/fl^ mice and TLR9 KO mice exhibited NASH component histology that was significantly less severe.

Pharmacological inhibition of TLR9 with a TLR7/9 oligonucleotide antagonist was tested next by Mehal’s group (101). Treatment with the TLR9 antagonist at 5mg/kg once weekly prevented NASH component histology, ALT elevations, and cytokine transcript levels when the treatment was administered concurrently with the HFD. The TLR9 antagonist also reversed NASH component histology, ALT elevations, and cytokine transcript levels (*pro-Il1b*, *Il6*, *Tnfa*) when administered after eight weeks of HFD once weekly for four weeks at the same dose while the mice remained on the HFD. The treatment had no effect on food intake.

Geoffrey Farrell’s lab in 2017 determined the effects of TLR9 deletion in NASH pathogenesis in mice fed an atherosclerotic diet (23% fat, 0.2% cholesterol w/w) (85). This diet contained a much lower level of cholesterol than the typical 1–1.25% cholesterol often used in atherosclerotic diets and no cholate. Therefore, this diet is also translationally relevant to human NASH. TLR9 KO mice fed an atherosclerotic diet gained weight proportionally, including visceral fat mass, to WT mice. There were no differences in serum insulin between WT and TLR9 KO, and fasting blood glucose in response to atherosclerotic diet feeding was similarly elevated. Serum total cholesterol and hepatic free cholesterol were lower than WT mice after atherosclerotic diet feeding. Intriguingly, ALT values failed to increase when the TLR9 knockouts were fed the atherosclerotic diet. WT mice fed the atherosclerotic diet had a significant decrease in serum adiponectin that was not observed in TLR9 KO mice.

The decrease in adiponectin in the diet-fed WT, but not the TLR9 KO mice, is fascinating because it immediately draws attention to dysfunctional TLR9 signaling in the primary compartment from which adipokine signaling originates, the adipose tissue. Adiponectin is an incredibly unique pleiotropic signaling molecule that modulates insulin target tissues (140, 141). In the liver, adiponectin decreases fat accumulation, glucose output, and inflammation; in adipose tissue, it functions to decrease inflammation and increase insulin-stimulated glucose uptake; and, in skeletal muscle, decreases fat accumulation, increases glucose uptake and energy expenditure. Adiponectin is not secreted by fat cells alone—hepatic stellate cells secrete adiponectin in the resting state, and activated stellate cells produce apoptosis when treated with adiponectin (142). Therefore, normally elevated adiponectin levels are a systemic regulator of inflammatory and metabolic intra- and inter-organ homeostasis (143). More on the relationship between TLR9 and adiponectin is discussed in the Sections titled *Adipose Tissue* and *Cutting the Endocrine Brakes on TLR9 Signaling*.

The group next looked at liver histological features of inflammation and fibrosis. Despite exhibiting equivalent levels of steatosis, hardly any inflammation was observed in the TLR9 KOs fed the atherosclerotic diet. Abundant inflammation was present in the diet-fed wild-type mice, a difference that was also supported by markers for matrix deposition and activated hepatic stellate cells.

On the molecular level, NF-κB and JNK activation was evident in hepatic nuclei, including hepatocyte nuclei, in WT atherosclerotic-diet fed mice. Similar activation in the TLR9 KO mice was absent. The observation of significantly less inflammatory recruitment (macrophages and neutrophils) in the diet-fed TLR9 KOs was consistent. The fatty livers of TLR9 KO mice expressed less MCP-1 and Th1 cytokines (*Mcp1*, *Tnf*, *iNos*, *Il-6*). Markers for Th2 cytokines (*Il-4*, *Ym1*, *Arg*, circulating IL-10) increased in both groups similarly, but the authors noted that the imbalance in Th1 cytokines shifted the cytokine balance to one of protection in the TLR9 KO mice.

Isolation of bone marrow macrophages and neutrophils from WT and TLR9 KO mice confirmed their findings. Macrophages from TLR9 KO mice lost the ability to generate TNF when cultured with necrotic hepatocyte media, and neutrophils exhibited less chemotaxis. Isolated macrophages from TLR9 KO mice did not lose the ability to respond to the M2 stimulatory IL-4.

To further investigate if TLR9 expressed on bone marrow-derived cells was essential for NASH pathogenesis in this model, they created chimeric WT mice with TLR9^-/-^ myeloid cells. When fed an atherosclerotic diet, the chimeric mice with TLR9^-/-^ myeloid cells had impaired levels of circulating pro-inflammatory cytokines compared with chimeric WT mice with WT-TLR9^+/+^ myeloid cells.

The group also found decreased cytochrome c in the liver lysates of atherosclerotic diet-fed TLR9 KO mice compared with WT mice. The explanation for the observation was unclear at the time, but now makes sense with the discovery three years later that an AMPK-caspase-6 regulated mechanism activates a feed-forward loop fueled by cytochrome c release resulting in hepatocyte death (139).

### Adipose Tissue

Nishimoto first reported that genetic ablation of TLR9 improves insulin resistance through decreased macrophage accumulation in adipose tissue at the European Society of Cardiology in 2013 (144). In the publication that followed, the group demonstrated in C57BL/6 mice on a HFD that obesity-related adipocyte degeneration causes the release of cell-free DNA (cfDNA) in the visceral adipocyte compartment (100). The release of cfDNA was associated with increased visceral adipose tissue (VAT) weight, but not liver weight. Compared with lean mice, the HFD enhanced adipocyte degeneration. Concurrently, TLR9 transcript by RT-PCR increased in the VAT and was dominant in the macrophage population of the VAT. Using transwell co-culture experiments, the group established that TLR9 activation by cfDNA released from degenerated adipocytes increased monocyte chemoattractant protein-1 (MCP-1) expression.

To determine if TLR9 promoted adipose tissue inflammation by accelerating macrophage infiltration into the tissue, they used WT and TLR9 KO mice. After 12 weeks of feeding, body weight, VAT weight, and food intake were similar between the two groups. Compared with the WT mice, the TLR9 knockouts showed reduced macrophage infiltration and reduced expression of MCP-1 and TNF-α. In epididymal fat tissue of TLR9 KO mice, data suggested the macrophages were M2 polarized, while in the WT mice, the macrophages were M1 polarized. Additionally, the VAT of the TLR9 KOs showed less NF-κB activation and better insulin sensitivity. Both adiponectin and PPARγ were significantly higher in the VAT of the TLR9 KO mice fed a HFD than the WT counterparts. The observations on adiponectin in this HFD model were consistent with the TLR9-KO mice fed the atherosclerotic diet mentioned in the previous section (85).

The creation of chimeric TLR9 knockout mice with the bone marrow of wild-type (*Tlr^+/+^)* mice demonstrated that chimeric mice have more macrophage infiltration into adipose tissue, higher levels of inflammatory molecules, higher NF-κB activation, and more insulin resistance compared with control animals on the same diet without TLR9 expressing bone marrow.

Administration of a TLR9 inhibitory oligonucleotide at approximately 5 mg/kg, three times a week, resulted in reduced accumulation of macrophages in adipose tissue and improved insulin resistance. The treatment also decreased the level of plasma triglycerides with no difference in food intake.

A group from the Diabetes Research Group at the Toronto General Research Institute observed that the adipocytes in the VAT compartment may not be the only source of TLR9-activating molecules (145). VAT macrophages were also observed to expel extracellular traps (ETs) composed of nucleic acids. HFD-fed mice had increased formation of ETs in VAT, and TLR9 KOs had fewer M1 macrophages, fewer crown-like structures, and improved glucose homeostasis and insulin signaling during HFD feeding. Despite no difference in body weight to WT controls, the TLR9 KO mice fed a HFD showed decreased liver weights and decreased hepatic steatosis. Detailed metabolic profiling demonstrated that TLR9 KO mice also had similar food intake, oxygen consumption, CO_2_ output, respiratory exchange ratio, and energy expenditure to WT control mice.

Plasmacytoid dendritic cells (pDCs) are nearly absent in VAT but are present in the liver, where they can lead to prolonged inflammation and hepatocyte damage. TLR9 KO mice fed a HFD had decreased numbers of pDCs in the liver. They identified IFNα as a possible agent of hepatic insulin resistance and noted the number of IFNα-positive pDCs was consistently decreased in TLR9 KO mice fed a HFD compared with wild type mice fed a HFD. Exogenous introduction of TLR9 agonist in 20-week-old NCD mice worsened glucose tolerance, increased the number of hepatic pDCs, and decreased the number of tolerogenic pDCs.

Finally, obese mice treated for three weeks with ~3.3 mg/kg/week TLR9 oligonucleotide antagonist had improved glucose tolerance and a tendency for lower fasting insulin compared with PBS-injected controls.

Unlike other reports of TLR9 KOs, Hong et al. reported that TLR9 deficiency accelerates HFD-induced weight gain, insulin resistance, and adipocyte dysfunction (146). The observation of similar food intake between the control and knockout group was the same as previous studies. It is unknown why this study is an outlier. However, one possible reason is that it is the one report employing TLR9 knockouts initially developed on a 129P2 background before being backcrossed to B6 [as the difference in weight between WT and TLR9 KO employing the same transgenic model was seemingly replicated in the recovery phase of a study performed by a different lab (147)]. In normal physiological conditions, TLR9 certainly has a protective role. The results of this TLR9 KO study may be more illustrative of the importance of TLR9’s protective function in normal physiology, but it is difficult to know without an investigation into how the methods differed between studies.

## Exacerbation of TLR9 Activation From Other Sources in NASH Pathogenesis

The stressed parenchyma of the liver and adipose compartments along with possible gut leakage are not the only source for TLR9-activating molecules. It was observed that lymphocytes (B cells, T cells, NK cells), as well as monocytes and neutrophils, can secrete mtDNA webs in response to CpG oligonucleotides (148). mtDNA webs are distinct from neutrophil extracellular traps, which are expelled genomic DNA complexed with antibacterial proteins (149). The secretion of mtDNA webs upon stimulus with CpG oligonucleotides was not dependent on TLR9, as targeted TLR9 inhibition or other techniques to prevent TLR9 endosomal signaling did not impact mtDNA secretion. This result seems to indicate the existence of at least one feed-forward loop to increase the amount of mtDNA present in the hepatic milieu, independent of TLR9.

A different group documented a TLR9-dependent negative feedback loop that limited neutrophil overactivation upon stimulation with mtDNA, which could putatively function in the same system as the feed-forward loop (150). TLR9 activation upregulated the main actor of the negative feedback loop, miR-223. miR-233 knockout mice were more susceptible to activation of inflammatory mediators and NF-κB by TLR9 agonists. The same group later observed that miR-223 is initially elevated in the hepatocytes of rodents on a HFD and in human NASH samples indicative of a protective function, but that expression levels likely deteriorate as NASH disease worsens and progresses to cirrhosis and hepatocellular cancer (151). Therefore, NASH disease progression could be modulated by the concurrent downregulation of miR-233 that allows TLR9-ligand-activated signals to increase (152–154).

Last, hepatic free cholesterol accumulation in the liver alters normal transport of cellular cargo, including endosomal TLR9. The association between the accumulation of hepatic free cholesterol and NAFLD and fibrosis is well characterized (155, 156). In hepatic sinusoidal endothelial cells, free cholesterol accumulation exacerbated TLR9 signaling in a model of acetaminophen (APAP)-induced liver injury in obese animals (157). APAP injury is directly tied to the release of mtDNA in mice and humans (158). The authors observed that elevated free cholesterol levels in endolysosomes impaired the trafficking of TLR9 from late endosomes to lysosomes *via* Rab7. TLR9 escaped degradation and accumulated, thus enhancing TLR9 signaling. In *Tlr9^-/-^* mice, the effects of increased intake of cholesterol on APAP injury disappeared. Treatment of mice with ~3.3 mg/kg of oligonucleotide TLR9 antagonist 4 h after APAP treatment significantly ameliorated cholesterol-loading induced TLR9 signaling.

Free cholesterol accumulation in hepatocytes is also a likely source for TLR9 activation. In free cholesterol loaded hepatocytes, HMGB1 was released into the culture medium (158). It had already been reported that HMGB1 is an important modulator of TLR9 activation by CpG containing DNA (159–161). Extracellular HMGB1 accelerates the formation of the CpG-DNA–TLR9 complex to lower the effective concentration of CpG DNA necessary for activating cellular responses.

In addition to the core function of sensing danger signals in the cell’s environment, TLR9 also senses mtDNA that originates from its own cell. In an *in vivo* model of mitochondrial dysfunction caused by deficiency of Opa1, a regulator of mitochondrial fusion and functional compartment formation (162), Opa-1 deficiency driven inflammation required mtDNA and was independent of cGAS. mtDNA ejected from the mitochondria was not present in the cytosol and was detected by TLR9 in the endosome. Incubation of the cells with a TLR9 oligonucleotide antagonist attenuated the expression of NF-κB genes. The exact mechanism of how TLR9 interacts with mtDNA from its own cell needs to be clarified. The authors speculate that the interaction could occur *via* mitochondrial-derived vesicles under conditions of stress.

## Evidence of TLR9 Activation and Integral Involvement in Human NASH Disease

The strongest data that ties TLR9 activation to NASH disease is data from the Sanyal lab that directly associates TLR9 activation with NASH and fibrosis disease severity in human patients. The authors analyzed hepatic gene expression and coordinately regulated pathways in disease and control cohorts characterized by biopsy across the full histological spectrum of NASH disease (163). Incredibly valuable was the availability of the supplementary data from this study on the Gene Set Variation Analysis (GSVA). The GSVA analysis allowed identifying specific pathways differentially regulated with increasing histological severity in NASH and NASH-associated fibrosis. In the GSVA analysis, most of the identified pathways that appear within the first several hundred pathways that meet the statistical threshold for discoveries by the false discovery rate approach are too broadly described to be specifically druggable (e.g., “Intrinsic Pathway for Apoptosis”, “Cellular Responses to External Stimuli”). Of the handful of pathways listed that are directly targetable, differential activation of TLR9 and TLR9 signaling and adaptor proteins are all significantly associated with NASH disease severity, all with adjusted p-values of <0.0001. The TLR9 cascade is also significantly associated with NASH-fibrosis severity with an adjusted p-value of 0.01. The GSVA analysis cannot distinguish between pathways that are drivers of progressive disease and pathways activated as secondary to disease severity. Therefore, the results must be placed in the appropriate context with interventional studies. In combination with the interventional studies described in the previous section titled *Liver* the GSVA offers compelling evidence from human biopsies that TLR9 signaling is directly associated with the severity of NASH disease.

There are naturally occurring loss-of-function variants of TLR9 (164). However, the variants may be too rare (< 1%) to test the hypothesis that they are protective in NASH. As the only known subjects of this rare variant have been detected in North-Western Europe, it may be worthwhile to investigate further the specific hypothesis in NASH and control databases that intersect with that geography (165).

Instead of investigating a loss-of-function population, Alegre et al. took the approach of identifying patients with matched parameters of metabolic dysfunction (including BMI, HOMA-IR, lipids), but who were diagnosed by biopsy with either simple steatosis or NASH (166). They focused on TLR9 expression in T cells, as intrahepatic T cells’ role in NASH progression was confirmed in several studies. They also investigated the T cell production of IFN-γ *via* activation of TLR9 in cells from the matched patient cohorts. T cell production of IFN-γ is critical for the differentiation of proinflammatory macrophages. They found that reduced expression of TLR9 in T cells, both hepatic and peripheral, was associated with lower liver necroinflammatory activity and fibrosis. When they co-stimulated T-cells *via* TLR9, the cells from the patients with simple steatosis produced a limited number of IFN-γ producing CD8+ T cells compared with the T cells from patients with NASH. They concluded that limited expression, or active downregulation, of TLR9 on T cells is protective. In turn, this would also favor the differentiation of anti-inflammatory (M2-polarized) Kupffer cells. Patients with NASH may have limited endogenous expression of TLR9 or failure of the downregulation mechanism. The observation is strikingly similar to TLR9 expression in surgical lung biopsies differentiating rapidly from slowly progressing forms of idiopathic pulmonary fibrosis (167). This single study does not parse correlation versus causation with a high enough level of evidence for generalization, but the results are intriguing. The study certainly supports the role of TLR9 activation on yet another type of immune cell that could be driving NASH disease.

## A Unified Theory: TLR9 Is More Than an Innocent Bystander in the Progression of NASH and NASH-Fibrosis

**Figure 2:**
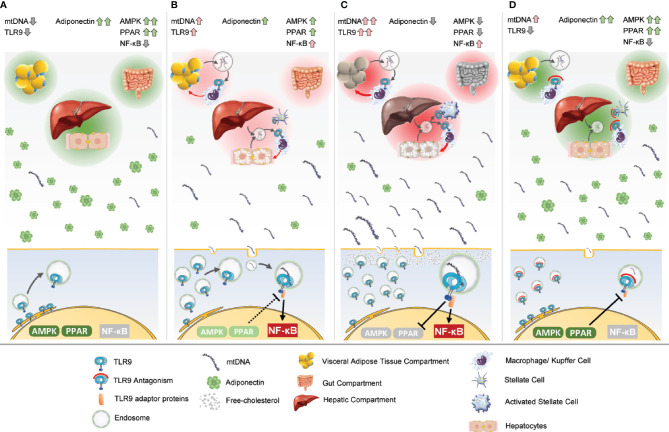
Unification of the theory of TLR9’s integral role in NASH pathogenesis. Three critical tissue compartments to NASH pathogenesis are pictured at the top of each frame, the adipose, gut, and liver. Endocrine and paracrine signals work at the compartment level and the level of the individual cell, pictured at the bottom. **(A)** The adipose, gut, and liver tissues are healthy and unstressed in a lean individual. Adiponectin levels are normally elevated. Transient elevations in mtDNA may occur in the course of normal physiology. PPAR is functioning normally, which dampens any transient TLR9 activation. TLR9 and AMPK are coupled so the cell can appropriately regulate energy expenditure if TLR9 is transiently activated, but in the resting state, most of the TLR9 is localized to the ER. NF-κB is not activated. **(B)** The adipose, gut, and liver tissues become increasingly stressed with overnutrition. Hepatosteatosis is evident. The secretion of mtDNA as response to the stress causes TLR9 upregulation. TLR9-dependent adipose infiltration and activation of macrophages and Kupffer cells occurs in the hepatic compartment. Gut leakage may allow bacterial product translocation into the portal circulation, either priming or activating TLR9 in the liver. A pro-inflammatory positive feedback paracrine loop forms between the infiltrating immune cells and the parenchyma. mtDNA levels increase in the systemic circulation. As adipocytes become more stressed, the levels of adiponectin decrease, causing AMPK activation to also decrease. Concurrently, increased TLR9 activation dampens PPAR activity. TLR9 activates pro-inflammatory NF-κB. **(C)** The various tissue compartments are even more stressed, and the TLR9-dependent pro-inflammatory positive feedback loop is robust. There are high levels of circulating mtDNA, and adiponectin levels are low. TLR9 activation may actively be suppressing PPAR activation, and adiponectin levels are too low to dampen TLR9 signaling. Free cholesterol present in the cell may further amplify TLR9 signaling. Stellate cells are activated in the liver, and hepatic fibrosis begins. **(D)** TLR9 antagonism prevents the inflammatory paracrine loop in the tissue compartments. Fibrosis is attenuated by TLR9 antagonism of stellate cell activation. PPAR signaling rises to dampen TLR9 activation as the system regains homeostatic function. Adiponectin works systemically to increase energy expenditure, decrease fat accumulation, and decrease inflammation and fibrosis.

Evidence suggests that two factors simultaneously contribute to NASH progression: elevations in circulating TLR9 agonists in response to organ stress because of overnutrition; and upregulation of the TLR9 receptor in both immune and non-immune cells. Most likely, the time-integrated exposure of chronic TLR9 activation across the liver, adipose, and gut drives progression of the disease over a period of years (**Figure 2**).

### A Healthy System

In normal physiology, TLR9 is expressed in both immune and non-immune cells to varying degrees and is localized to the endoplasmic reticulum on resting cells. In the liver, Kupffer cells are M2 polarized, which TLR9 may even facilitate in normal physiological states in conjunction with normal PPAR functioning (168, 169). Hepatic dendritic cells are tolerogenic and immature. Other surveilling immune cells, such as neutrophils and lymphocytes (liver natural killer cells) are ready to respond if attracted from the periphery or in the early defense against pathogens (170).

Outside the liver, the gut is healthy and under TLR9 homeostatic control. The visceral adipose tissue surrounding the liver is healthy and circulating adiponectin levels act on the liver and systemically to control the metabolism of glucose and lipids by stimulating AMPK and PPARα (171). In hepatocytes, AMPK activity is acting to sustain a healthy hepatic parenchyma (139).

### Primed for NASH by the Stresses of Overnutrition

In states of obesity and overnutrition, the liver is under a considerable amount of immune stress even if hepatosteatosis is not harmful *per se*. Liver fat flux is relatively fast, hepatic steatosis is often self-limited, and hepatic energy metabolism in patients newly diagnosed with fatty liver is not different than controls (172, 173). However, there is evidence that the complement system is activated in obese patients with steatosis without NASH (174, 175). NASH may play out on the stage of the liver by virtue of being a first-pass organ. The liver is exposed to the highest concentration of TLR9 agonists from the portal circulation. NAFLD, in its subclinical phase in states of obesity, is thought to condition hepatic cells for the transition from a normal physiological state to a disease state.

It could be that TLR9 activation in Kupffer cells and other hepatic cells are primed by LPS leakage from the gut. *In vitro*, it was demonstrated that LPS-pretreated mouse bone marrow-derived macrophages produced significantly more TNF and IL-6 when stimulated with CpG DNA (176). The effects were still evident 12 h post-LPS treatment, meaning it was not a requirement that the LPS and CpG signals be administered at the same time for the signal amplification to occur. The levels of LPS used in the *in vitro* study were consistent with levels observed in NASH subjects (177, 178). Recent evidence even supports intrahepatic residence of bacteria that could also be priming TLR9 in the liver (179).

These studies are consistent with LPS’ downregulating effects on IL-1R8, an IL-1 receptor family member that can put the brakes on TLR9 signaling (180). It is unclear in what subset of patients LPS may incrementally contribute to TLR9 priming. In a study of pediatric patients who were either obese or had biopsy-proven NASH, endotoxin levels were increased in only 42.1% (8 of 19) in the NASH group (181). The reports on the association of endotoxemia with NASH histological severity is mixed (182, 183).

Gao et al. reported that mtDNA from mice fed a HFD, when combined with LPS stimulation, caused the release of significantly higher amounts of pro-inflammatory IL-33 from cultured bone-marrow-derived macrophages than LPS alone (105). The amount of IL-33 released into the media when mtDNA from mice on a chow diet was combined with LPS stimulus was no greater than LPS stimulus alone. Production of IL-33 was reduced by more than 50% by treatment with either a TLR9 oligonucleotide antagonist or TLR9 knockdown with siRNA.

By the time hepatosteatosis is evident, the visceral adipose tissue surrounding the liver is surely stressed and in a state of metainflammation (184). Circulating adiponectin levels are reduced (185). As the adipose inflammation worsens, adipocytes exhibit necrotic-like abnormalities that trigger the recruitment of inflammatory cells such as macrophages. mtDNA is released by the stressed adipocytes as a damage-associated molecule pattern (DAMP), which not only exacerbates inflammation at the local tissue level but is released into the circulation. TLR9 expression increases in the visceral adipose tissue (84).

At the same time TLR9 activation priming is occurring, subclinical conditioning during NAFLD may also be tipping the balance towards favoring greater levels of TLR9 activation. One of those regulatory mechanisms involves the peroxisome proliferators-activated receptors (PPARs). The PPARs act as fatty acid sensors and act as master regulators of metabolism, energy homeostasis, and inflammation (186, 187). Regulation of the various PPAR subfamilies is complex, but PPAR silencing is a common observation in obesity (188–193). In a morbidly obese population, a high fat meal resulted in a significant decrease of PPARγ mRNA expression (194). The ratio of a naturally occurring dominant-negative splice isoform PPARγΔ5 to PPARγ in humans correlates with BMI in overweight or obese and diabetic patients (195). PPARα transcription and immunofluorescence staining in liver tissue were significantly reduced in a cohort of Chinese NAFLD patients that was height- and weight-matched to a healthy cohort (196).

The significance of normal PPAR functioning is that it inhibits TLR9 signaling. The PPARγ-activating thiazolidinedione (TZD) troglitazone, when added to cultured peripheral blood adhering monocytes stimulated with a TLR9 family member ligand, prevented IL-6 release and decreased stimulatory capacity (197). TLR9 family member-associated MAPK signaling (p38 and p42) is significantly blunted when treated with PPARγ’s natural ligand, 15-deoxy- PGJ2. In fact, the connection between PPARs and TLRs is well documented (198). This is also consistent with the transrepression of TLR9 signaling by a different member of the nuclear-receptor family, the glucocorticoid receptor (199). NAFLD has been associated with signaling changes that reduce glucocorticoid receptor signaling (200, 201). Indeed, impairment of glucocorticoid receptor signaling causes steatosis, and if restored, reverses NAFLD in mice (202, 203). That the glucocorticoid receptor transrepresses TLR9 signaling is consistent with a model wherein nuclear receptor families, like PPARs, are silenced, which removes the brakes from and augments TLR9 signaling.

TLR4, which functions through a common adaptor protein with TLR9 and similarly activates NF-κB, inhibits PPARγ mRNA synthesis when activated *via* a negative feedback loop involving NF-κB (204). It seems likely that TLR9-mediated NF-κB activation also inhibits PPAR mRNA synthesis.

Augmented TLR9 signaling has consequences in both immune and non-immune cells. In both the liver and adipose compartments, TLR9 activation on immune cells causes the release of cytokines and chemokines (205). TLR9 dependent overactivation of the immune component, particularly the activation of hepatic stellate cells, potentiates fibrosis (78). On non-immune cells, such as parenchymal hepatocytes and adipocytes, dysfunctional TLR9 signaling may be directly tied to disturbed energy homeostasis.

TLR9 signaling is directly coupled to the master regulator of energy homeostasis, AMPK (206). In the context of exercise and glucose starvation under normal physiological conditions, TLR9 is required to activate AMPK *via* an association with beclin1 and simultaneous TLR9 binding to endogenous mtDNA (35). They found that TLR9 expression is also required for AMPK-regulated effects on glucose metabolism during the stress of acute exercise. Therefore, the data suggest that under normal physiological conditions, transient TLR9 activation by mtDNA to activate AMPK is part of a normal physiological process. A biological mechanism that controls the transient activation of AMPK in normal physiology may be important, as it was demonstrated that a constitutively activated AMPK in mice induced obesity and reduced beta cell function (207).

The connection between beclin1, a regulator of autophagy, and TLR9 was the result of a screen to identify proteins that interacted with a region of beclin1 that is sufficient to promote autophagy when introduced exogenously (208). Beclin1 is a key part of phosphatidylinositol 3-kinase complex (PI3KC3) signaling in the endosome (209). The association with beclin1 led the group to find that TLR9 is also required for the association of beclin1 and UVRAG. UVRAG is another key component of the Class II phosphatidylinositol 3-kinase complex (PI3KC3-C2) crucial for endosomal signaling. The conclusion was that TLR9 regulates the assembly of PI3KC3-C2, which in turn regulates AMPK activity.

In a separate stream of work, Nemazanyy et al. found that a PI3KC3 complex containing UVRAG was a key node in the negative feedback inhibition of metabolic signaling (210). The PI3KC3 complex they investigated contained UVRAG, beclin1, and Vps15, common core components to Class II and Class III PI3KC3 (211). Vps15 acts as the regulatory subunit of PI3KC3. Through experiments that interfered with the expression of Vps15, they made the novel finding that PI3KC3 containing UVRAG and beclin1 had a previously unappreciated role in whole body nutrient homeostasis and control of metabolic adaptation. *In vivo¸* hepatic downregulation of Vps15 significantly improved glucose tolerance in *ob/ob* mice, decreased liver steatosis, and decreased hepatic triglyceride levels without the observation of a change in plasma metabolite levels. The findings were consistent with reports that established Vps15 as part of a complex thought to integrate environmental cues through an AMPK-dependent mechanism (212). Changing AMPK cellular localization, ordinarily present in both the cytoplasm and nucleus but in periods of stress (including cellular oxidants) shifts to the nucleus, is surely a part of this story (213). That TLR9 is a likely component, even rate-limiting component, of the PI3KC3 complex puts the receptor front and center in metabolic regulation through environmental cues.

One possibility is that chronic stimulation of TLR9 by endogenous mtDNA in states of overnutrition leads to the constitutive negative feedback of metabolic or autophagy signals through TLR9’s regulation of PI3KC3 assembly. This is consistent with AMPK downregulation and its role in liver damage in NASH (139, 214, 215). The regulation of expression of the TLR9-interacting protein beclin1 is significantly different in lean and obese states. When HFD-fed obese and lean mice are maintained on a 15 day 40% caloric restriction, then returned to *ad libitum* feeding on their original diets, refeeding led to a greater than 2-fold increase in beclin1 in the visceral adipose tissue in the obese mice whereas an 80% reduction in beclin1 was observed in the lean mice (216). Similarly, in humans, beclin expression was significantly higher in the adipose tissue of both non-diabetic and diabetic obese subjects than lean controls. Following gastric bypass, a significant drop in the expression of beclin was observed in both the obese groups. It is unknown how Vps15 responds in states of overnutrition. However, the discoveries that deficiency in other adaptor protein components of the Beclin1-Vps15 complexes in the AMPK pathway leads to lipid accumulation in the liver echoes the importance of this regulatory system to which TLR9 belongs (217). The two-way regulation of PI3KC3 complexes by AMPK is complex (212), and more investigation into the role TLR9 and TLR9 stimulation in both lean and obese states play in this pathway is needed.

Other mechanisms that modulate TLR9’s involvement between inflammation and energy modulation in non-immune cells have also been reported. In normal physiological systems, it was reported that TLR9 reduces energy substrates (intracellular ATP) in stressed cardiomyocytes by activating AMPK (34). AMPK activation may be turned off in disease-state TLR9 activation by the pivotal switch, Unc93b1. The shRNA-mediated knockdown of *Unc93b1* in macrophages could replicate the AMPK activation observed in the cardiomyocytes instead of observing the more prototypical inflammatory response. Conversely, overexpression of *Unc93b1* in cardiomyocytes reduced TLR9-induced AMPK activation and activated inflammatory signaling. *Unc93b1* overexpression also transformed the trafficking of both TLR9 and endocytosed CpG DNA so the agonist and cognate receptor could successfully meet in the endosome. They validated the results in a completely different kind of non-immune neuroblastoma cell line. Therefore, Unc93b1 may be an additional regulatory component in the switch from TLR9 activation of AMPK out of self-protection, to AMPK silencing in a disease state. Indeed, in the severely obese, UNC93B is upregulated (Lawless and Greene 2012, Clayton 2016). While there is no reported direct interaction between UNC93B1 and regulation of the PI3KC3 complex, they have shared involvement in TLR9 stabilization, endosomal transport, and modulation of AMPK (218). The possible decoupling of TLR9 and AMPK through TLR9 overaction needs to be investigated.

### Cutting the Endocrine Brakes on TLR9 Signaling

Impaired AMPK activation is intimately tied to another impaired global signaling system in NASH, the adipokine adiponectin. Adiponectin receptor activation increases AMPK and PPARs, resulting in increased fatty acid oxidation and glucose utilization (219, 220). Adiponectin also has anti-inflammatory properties targeted toward both hepatic and immune cells (221). The hormone targets the key organs involved in metabolic regulation, including the liver, heart, pancreatic β cells, kidney, and skeletal muscle. Scherer’s landmark discovery in 1995 of adiponectin marked the beginning of understanding the hormone’s intimate ties to the metabolic syndrome (222–224). Metabolic syndrome is strongly associated with decreased levels of circulating adiponectin, “hypoadiponectinemia”. A number of studies, too many for inclusive citation here, have demonstrated the strong association between NASH and decreased levels of circulating adiponectin in both adults and pediatrics (225–228). NASH patients have lower circulating adiponectin levels than patients with NAFLD (229).

Adiponectin is an inhibitor of TLR9 signaling. Yamaguchi et al. found that pretreatment of macrophages with globular adiponectin significantly inhibited NF-κB activation after CpG DNA stimulation (230). The adiponectin levels that suppressed TLR9 activation *in vitro* were consistent with those observed in metabolically healthy, non-obese Caucasian subjects and also Asian subjects without NAFLD (231, 232). Therefore, adiponectin is yet another braking mechanism for TLR9 activation that is removed in subjects with NASH. Studies to determine a direct association between circulating adiponectin levels, TLR9 activation and the severity of NASH and fibrosis should be pursued.

### Convergence to NASH and NASH-Associated Fibrosis

By this moment in time, the totality of the literature suggests a robust positive feedback loop exists between the liver, gut, and adipose compartments involving circulating mtDNA triggering TLR9-dependent inflammatory activation in immune cells and upregulation of TLR9 in non-immune cells. The putative decoupling of TLR9 from AMPK may further dysregulate macrophage polarization in adipose tissue, further amplifying the paracrine loop between adipocytes and infiltrating macrophages (233, 234). Outside the liver-VAT-gut axis, TLR9 overactivation may induce podocyte apoptosis, accelerating insulin resistance and leading to the metabolic syndrome (235, 236).

Concurrent with the upregulation of TLR9 across various tissues, the molecular brakes tempering TLR9 activation have been removed, such as PPAR signaling, which forms a feed-forward loop with aberrant TLR9 trafficking to result in TLR9 signal amplification. The inflammatory milieu attracts neutrophils into the liver, and subsequent neutrophil overaction leads to the secretion of mtDNA webs.

The positive feedback loop continues to exacerbate hepatocyte cell degeneration with aberrant AMPK signaling leading to hepatocyte cell death (237, 238). Apoptotic hepatocyte DNA provides both a stop signal and stationary phenotype‐associated up‐regulation of collagen, both dependent on TLR9, in stellate cells (239). Gabele demonstrated that CpG stimulation of both human and murine hepatic stellate cells increases levels of the profibrogenic chemokine monocyte chemotactic protein 1 (MCP-1) and that TLR9^-/-^ rodents had significantly less MCP-1 and α1(I) collagen mRNA expression, and less fibrosis by histology when challenged with bile duct ligation (78). Hypoadiponectinemia makes stellate cells more susceptible to activation (240).

Evidence supports that mtDNA from degenerated, injured, or apoptotic hepatocytes leads to hepatic fibrosis. Ballooned hepatocyte cells are independently associated with both sinusoidal fibrosis and perivenular fibrosis in NASH patients (241). Popov demonstrated that the failure to clear dead hepatocytes by persistent macrophage infiltrates (impaired efferocytosis) led to fibrosis in a thioacetamide (TAA)-induced model of fibrosis in mice (107). In the TAA model, they observed a 3-fold elevation of circulating mtDNA in serum levels post-TAA treatment in a mouse strain that was particularly susceptible to fibrosis because of impaired efferocytosis. The same elevation in circulating mtDNA post-treatment was not observed in a mouse strain resistant to TAA-induced fibrosis that also had functioning efferocytosis. The group could recapitulate the severity of fibrosis of the susceptible mice in the resistant mice by injecting the resistant mice with mtDNA post-TAA treatment. The injection of mtDNA in the resistant mice mimicked the prolonged exposure by susceptible mice to circulating mtDNA. The “resistant” mice developed significant liver fibrosis.

When Popov isolated hepatic stellate cells *in vitro* and treated them with increasing doses of purified mtDNA from hepatocytes, dose-dependent changes were observed in morphology characteristic of activation, increased proliferation, and profibrogenic gene expression. Therefore, stellate cell fibrogenesis seems directly linked to circulating mtDNA. TLR9 on stellate cells may be the primary receptor for detecting the prolonged elevations in circulating mtDNA in progressive NASH, and hepatic fibrogenesis advances.

## Therapeutic Perspective

The cumulative data supports that TLR9 antagonism is a promising therapeutic approach to treating NASH. TLR9’s place at the intersection of metabolism and inflammation is an important node for promising therapeutic intervention. Any therapy developed for this indication needs to be safe and well-tolerated across a broad population of people, and the safety database for TLR9 antagonism is supportive (242).

The most promising cornerstone strategies for NASH are likely those with comprehensive biological activity that match the multifactorial pathogenesis of NASH disease. Advances in FGF21 and GLP-1 analogs with clinical action on body weight, lipids, and adipokines are particularly interesting (243, 244). Both strategies fall under the category of metabolic agonists. Other late-stage pipeline candidates address narrower biology and disease nodes less proximal to positive energy balance, such as inflammation and cellular stress (ASK1 and CCR2/5 antagonists), lipid metabolism (thyroid hormone analogs), and *de novo* lipogenesis (FXR agonists) (245). The landscape covers the various therapeutic hypotheses of the main pathogenic mechanisms of NASH and NASH-associated fibrosis. The question remains what level of information exists to indicate that a patient will have a superior benefit-risk to a prescribed therapy.

The multi-factorial nature of NASH pathogenesis has made the afflicted population difficult to subset. The clinical interest in finding subsets of a disease population is in selecting patients more likely to respond to a therapeutic strategy, one component of precision medicine (246). That patients more likely to respond to TLR9 antagonism may be identified by measurement of circulating levels of TLR9 agonist or by observation of TLR9 (over)-expression in hepatic or adipose tissue, or by a combination of both, could allow for a degree of precision medicine in such a complex disease.

However, the data supports that circulating mtDNA levels are significantly elevated in the vast majority of NASH and NASH-fibrosis patients who are obese, and TLR9 activation is significantly associated with disease severity in unenriched cohorts of NASH and NASH-fibrosis patients. There may not be a bright line separating minimal and maximal responders—it may be more of a “ragged edge,” as Fleck calls it (247). The existing data suggest that TLR9 antagonism would benefit the broad majority of patients with NASH and NASH-fibrosis. The hypothesis merits clinical testing.

One of the primary advantages of TLR9 antagonism in NASH may be in the therapeutic index. Upon reviewing the research, we see that many of the molecular players coupled to TLR9 signaling are therapeutic targets in the current pipeline for NASH and NASH-fibrosis (248, 249). PPAR and AMPK signaling are compelling targets. While targeting those pathways with molecular agonists have shown effectiveness, they come with questionable safety profiles for use in broad populations (250, 251). Further, these molecular agonists are small molecules with systemic bioavailability, and therefore have pharmacologic (and potentially toxicologic) effects on critical organs not fundamental to NASH and NASH-fibrosis pathogenesis. The TLR9 antagonists used most often in the studies described here are oligonucleotide antagonists. The liver-gut-adipose-centric bioavailability of this mode of delivering TLR9 antagonism would seem to have an advantage in keeping the highest free drug concentrations limited to those organs most involved in the pathogenesis of NASH disease (252).

While adiponectin is a tempting target, there is no current strategy that allows for the direct drugging for adiponectin elevation (253). Adiponectin elevations secondary to other drug targets have proven clinically impactful. Observations of adiponectin elevations upon pharmacological PPARγ activation go back to 2002 (254). Merck Research Laboratories was quick to use adiponectin elevations as an example of a “putative biomarker” for PPARγ activity even in the nascent stage of biomarker application to drug development (255). Later, Cusi and colleagues demonstrated in clinical trials of the PPARγ activator pioglitazone that adiponectin elevation is the best predictor of histologic response in NASH and fibrosis (256, 257). Clinically relevant adiponectin elevations secondary to TLR9 antagonism would be a significant therapeutic advance.

Finally, TLR9 antagonism is compelling as a therapeutic strategy in NASH because it targets a positive feedback loop dependent on a chronic disease-specific signal, circulating mtDNA. Allowing the system to reset by pharmacologically returning to a more homeostatic state of inflammation and metabolism, one without chronic activation of TLR9 by mtDNA, is a plausible strategy for efficacious therapy. Antagonizing a disease-specific signal limits the safety liabilities associated with metabolic agonists.

## Conclusion

The critical mass of research supporting TLR9’s importance in the pathogenesis of NASH and NASH-associated fibrosis includes an integral role in the inflammatory process that fuels NASH, as well as a metabolic one. Elevated levels of circulating mtDNA in patients with NASH and NASH-associated fibrosis, along with the association between TLR9 pathway activation and NASH disease severity, is strongly suggestive when combined with the mechanistic animal models of disease. TLR9’s role in hypoadiponectinemia has implications for insulin sensitive tissues throughout the body. The evidence suggests that TLR9 functions as a critical node that modulates at least three master regulators of NASH pathogenesis: AMPK, PPAR, and NF-κB.

Much more is known about TLR9’s role in the inflammatory process than in dysregulated metabolism. There are undoubtedly unexplored research areas in how TLR9 coordinates the PI3KC complex that could prove valuable in elucidating new therapeutic targets or strategies to target TLR9 signaling. The evidence supports that TLR9 is an important node in the inflammatory and dysfunctional metabolic components of NASH and NASH-associated fibrosis. Targeting TLR9 in NASH may prove an efficacious clinical strategy for a disease that is still an unmet medical need for a large fraction of the population.

## Author Contributions

CS conceived, summarized the main conceptual ideas, made intellectual contributions, prepared the figures, and wrote the manuscript.

## Funding

No payment has been offered or will be offered to the author for authoring this publication and no compensation will be provided for the time he spent on the publication development. Structured BioEquity (SBE) funded CS’s participation as an Organizer of the 2019 Keystone Symposia Integrated Pathways of Disease in NASH and NAFLD.

## Conflict of Interest

CS is the founder of the company Avogadro Development Corp that has research and development interests in TLR9 antagonism in NASH.
